# Supplementary material for: Serum Phytosterols Are Not Associated with Inflammatory Markers in Two Cross-Sectional, Swiss Population-Based Studies (The CoLaus|PsyCoLaus Study)
Source: Nutrients. 2022 Jun 16;14(12):2500. doi: 10.3390/nu14122500 (PMC9229848; doi:10.3390/nu14122500)
Supplement: Supplementary file 1 [file nutrients-14-02500-s001.zip › nutrients-1767653-supplementary.pdf]

Supplemental material

Table S1: correlation coefficients between sterols and sterol ratios and inflammatory markers, first (2009-2012) and second (2014-2017) follow-ups, CoLaus|PsyCoLaus study, Lausanne, Switzerland.

	CRP		IL-6		TNF-α	
	**First**	**Second**	**First**	**Second**	**First**	**Second**
Cholesterol absorption						
Cholestanol [mg/dl]	**-0.114**	-0.081	**-0.090**	**-0.244**	**-0.088**	**-0.131**
Cholesterol synthesis						
Lathosterol [mg/dl]	**0.114**	0.018	0.010	-0.049	0.017	-0.002
Desmosterol [mg/dl]	**0.080**	-0.004	0.036	**-0.125**	0.031	-0.084
Lanosterol [μg/dl]	**0.109**	-0.032	0.044	**-0.122**	-0.008	-0.022
Dihydro-lanosterol [μg/dl]	0.052	-0.016	0.069	0.028	-0.033	0.014
Vegetal origin						
Campesterol [mg/dl]	**-0.118**	**-0.123**	-0.023	**-0.143**	-0.030	**-0.105**
Sitosterol [mg/dl]	**-0.130**	**-0.155**	0.001	**-0.132**	0.007	-0.083
Brassicasterol [μg/dl]	**-0.060**	**-0.091**	0.003	**-0.094**	-0.016	**-0.139**
Sitostanol [μg/dl]	-0.072	**-0.095**	0.057	**-0.093**	0.015	**-0.097**
Campestanol [μg/dl]	-0.089	**-0.096**	0.003	-0.085	-0.062	-0.084
Stigmasterol [μg/dl]	-0.025	**-0.117**	0.078	0.000	-0.013	-0.016
Ratios						
Cholestanol-to-TC ratio	**-0.111**	-0.004	-0.023	**-0.165**	-0.022	**-0.094**
Synthesis markers-to-TC ratio	**0.135**	0.035	**0.075**	-0.038	0.024	0.010
Campesterol-to-cholestanol ratio	-0.057	**-0.094**	0.036	-0.051	0.018	-0.064
Campesterol-to-TC ratio (100x)	**-0.108**	-0.077	0.007	**-0.100**	-0.004	-0.083
Stigmasterol-to-TC ratio	-0.019	-0.074	**0.108**	0.055	0.017	0.005
Sitosterol-to-TC ratio	**-0.115**	**-0.103**	0.041	-0.083	0.044	-0.067
Phytosterols-to-TC ratio	-0.049	-0.069	**0.104**	0.036	0.037	-0.017

Results are expressed as Spearman nonparametric correlation coefficient. Significant ($p<0.05$) results are indicated in bold and with a grey background

Table S2. Robust regression between inflammatory markers and serum sterol levels and ratios, first (2009-2012) and second (2014-2017) follow-ups, CoLaus|PsyCoLaus study, Lausanne, Switzerland.

	CRP		IL-6		TNF-α	
	**FU1**	**FU2**	**FU1**	**FU2**	**FU1**	**FU2**
**Cholesterol absorption**						
Cholestanol [mg/dl]	-0.571	-0.636	-0.629	**-0.549**	**-3.365**	**-1.297**
	(-1.606 ; 0.464)	(-1.529 ; 0.257)	(-3.342 ; 2.085)	**(-0.803 ; -0.295)**	**(-6.504 ; -0.225)**	**(-2.173 ; -0.420)**
**Cholesterol synthesis**						
Lathosterol [mg/dl]	**1.392**	-0.266	0.054	0.042	1.516	-0.017
	**(0.511 ; 2.272)**	(-1.302 ; 0.769)	(-2.248 ; 2.356)	(-0.113 ; 0.196)	(-1.164 ; 4.196)	(-0.545 ; 0.511)
Desmosterol [mg/dl]	-0.135	-0.188	-0.888	**-0.230**	0.438	-0.744
	(-0.824 ; 0.555)	(-0.997 ; 0.621)	(-2.668 ; 0.891)	**(-0.471 ; 0.011)**	(-1.636 ; 2.512)	(-1.572 ; 0.084)
Lanosterol [µg/dl]	**0.009**	0.009	0.005	-0.004	0.003	0.004
	**(0.002 ; 0.016)**	(-0.009 ; 0.027)	(-0.013 ; 0.023)	(-0.009 ; 0.001)	(-0.019 ; 0.024)	(-0.014 ; 0.022)
Dihydro-lanosterol [µg/dl]	0.016	**0.403**	**0.109**	0.063	-0.030	0.178
	(-0.014 ; 0.045)	**(0.040 ; 0.766)**	**(0.032 ; 0.186)**	(-0.043 ; 0.170)	(-0.119 ; 0.060)	(-0.189 ; 0.544)
**Vegetal origin**						
Campesterol [mg/dl]	0.142	-0.505	0.253	-0.140	-0.895	**-0.888**
	(-0.368 ; 0.651)	(-1.174 ; 0.163)	(-1.060 ; 1.566)	(-0.338 ; 0.058)	(-2.422 ; 0.633)	**(-1.555 ; -0.221)**
Sitosterol [mg/dl]	-0.141	-0.691	1.385	-0.182	-0.155	**-0.706**
	(-0.928 ; 0.646)	(-1.553 ; 0.170)	(-0.642 ; 3.412)	(-0.438 ; 0.074)	(-2.526 ; 2.216)	**(-1.580 ; 0.167)**
Brassicasterol [µg/dl]	0.006	-0.008	0.015	-0.002	-0.005	**-0.019**
	(-0.002 ; 0.015)	(-0.018 ; 0.002)	(-0.008 ; 0.038)	(-0.005 ; 0.001)	(-0.032 ; 0.022)	**(-0.029 ; -0.009)**
Sitostanol [µg/dl]	-0.009	-0.008	0.043	-0.012	0.042	-0.042
	(-0.033 ; 0.015)	(-0.055 ; 0.040)	(-0.019 ; 0.106)	(-0.026 ; 0.003)	(-0.031 ; 0.116)	(-0.091 ; 0.007)
Campestanol [µg/dl]	-0.013	-0.055	-0.001	-0.012	-0.036	-0.062
	(-0.038 ; 0.011)	(-0.123 ; 0.013)	(-0.065 ; 0.063)	(-0.032 ; 0.008)	(-0.111 ; 0.039)	(-0.132 ; 0.007)
Stigmasterol [µg/dl]	0.009	-0.010	**0.106**	0.004	-0.037	0.012
	(-0.015 ; 0.034)	(-0.04 ; 0.020)	**(0.041 ; 0.170)**	(-0.004 ; 0.013)	(-0.111 ; 0.038)	(-0.018 ; 0.042)
**Ratios**						

Cholestanol-to-TC ratio	-0.132	-0.042	0.292	**-0.129**	-0.580	**-0.381**
	(-0.416 ; 0.152)	(-0.236 ; 0.151)	(-0.441 ; 1.024)	**(-0.184 ; -0.074)**	(-1.438 ; 0.278)	**(-0.573 ; -0.190)**
Synthesis markers-to-TC ratio	**0.002**	**0.004**	0.003	-0.001	0.002	0.000
	**(0.001 ; 0.004)**	**(0.001 ; 0.008)**	(-0.001 ; 0.007)	(-0.002 ; 0)	(-0.002 ; 0.007)	(-0.004 ; 0.004)
Campesterol-to-cholestanol ratio	0.045	-0.119	0.154	0.006	0.019	-0.268
	(-0.140 ; 0.230)	(-0.409 ; 0.172)	(-0.322 ; 0.631)	(-0.080 ; 0.092)	(-0.538 ; 0.575)	(-0.561 ; 0.026)
Campesterol-to-TC ratio	0.045	-0.078	0.168	-0.029	-0.127	**-0.185**
(100x)	(-0.072 ; 0.162)	(-0.206 ; 0.050)	(-0.134 ; 0.470)	(-0.067 ; 0.009)	(-0.480 ; 0.226)	**(-0.313 ; -0.058)**
Stigmasterol-to-TC ratio	0.003	0.000	**0.033**	0.001	-0.003	0.001
	(-0.003 ; 0.008)	(-0.006 ; 0.006)	**(0.018 ; 0.048)**	(-0.001 ; 0.002)	(-0.02 ; 0.014)	(-0.005 ; 0.007)
Sitosterol-to-TC ratio	-0.024	-0.105	**0.532**	-0.039	0.186	-0.161
	(-0.207 ; 0.158)	(-0.269 ; 0.058)	**(0.062 ; 1.003)**	(-0.088 ; 0.010)	(-0.365 ; 0.738)	(-0.326 ; 0.004)
Phytosterols-to-TC ratio	0.000	0.001	**0.014**	0.000	0.005	-0.002
	(-0.003 ; 0.003)	(-0.003 ; 0.005)	**(0.007 ; 0.022)**	(-0.002 ; 0.001)	(-0.005 ; 0.014)	(-0.006 ; 0.002)

CRP, C-reactive protein; FU, follow-up; IL-6, interleukin 6; TNF-α, tumor necrosis factor alpha. Results are expressed as slope and (95% confidence interval). Statistical analysis conducted by robust regression adjusting for age (continuous), sex (male, female), BMI (continuous), metformin (yes, no) and statin use (yes, no). Significant ($p<0.05$) associations are indicated in bold.
